# A safe and effective dose of cisplatin in hepatic arterial infusion chemotherapy for hepatocellular carcinoma

**DOI:** 10.1002/cam4.55

**Published:** 2013-02-03

**Authors:** Akihiko Osaki, Takeshi Suda, Kenya Kamimura, Atsunori Tsuchiya, Yasushi Tamura, Masaaki Takamura, Masato Igarashi, Hirokazu Kawai, Satoshi Yamagiwa, Yutaka Aoyagi

**Affiliations:** 1 Division of Gastroenterology and Hepatology, Graduate School of Medical and Dental Sciences, Niigata UniversityNiigata, Niigata 951-8510, Japan; 2 Division of Gastroenterology and Hepatology, Niigata University Medical and Dental HospitalNiigata, Niigata 951-8520, Japan

**Keywords:** Cisplatin, dose recommendation, hepatic arterial infusion chemotherapy, hepatocellular carcinoma

## Abstract

Cisplatin (CDDP) is an anticancer agent that is commonly used in hepatic arterial infusion (HAI) chemotherapy for hepatocellular carcinoma (HCC). This study aimed to clarify the safe and effective dose of CDDP in HAI for HCC. The hypervascular area was measured in 42 HCCs before and after HAI with CDDP. Serum platinum concentration was quantified in the peripheral and/or middle hepatic veins by atomic absorption spectrometry. The relation between the HCC response and CDDP dose was statistically analyzed. The multiple HCC nodules in an individual case generally demonstrated the same response to CDDP. The free-platinum concentration stayed relatively constant in the hepatic vein during HAI followed by a rapid decline, while total-platinum gradually increased then slowly disappeared over several days. After CDDP-HAI, 15 HCCs shrunk and 27 HCCs grew. The reduction rate in the shrunken nodules was tended to be correlated with CDDP dose after standardization with the target liver volume. On the other hand, the growth rate of the enlarged HCCs was significantly correlated with CDDP dose after normalization with creatinine clearance. These data support a recommendation of CDDP-HAI infusion where the amount of CDDP (mg) administered is less than patient creatinine clearance (mL/min/1.73 m^2^) upon an assumption of HCC doubling time of 90 days, and the targeted liver is smaller than 200 times the CDDP dose (mg). A further analysis is required to define appropriate injection speeds.

## Introduction

Hepatocellular carcinoma (HCC) is the sixth-most common human cancer in the world and often has a poor prognosis [1]. Because functional liver reserve has an impact on patient prognosis that is similar to anatomical cancer extent [2,3], hepatic arterial infusion (HAI) chemotherapy is a promising therapeutic option, especially in cases with diminished functional liver reserve, as it does not physically deteriorate residual liver function as do treatments like surgical resection, radiofrequency ablation, or transcatheter hepatic arterial chemoembolization. HAI using cisplatin (CDDP-HAI) is especially preferable, as CDDP is mainly excreted from the kidneys, and the dose-limiting toxicities are nephron-, neuro-, and hematological in nature and, as such, rarely severely burden hepatocytes [4,5].

Cisplatin exerts its anticancer effects mainly through a direct chemical reaction with N-7 of guanine or adenine forming intra and inter strand covalent bonds with DNA [6–8]. Court et al. [9] reported that approximately half of the ^195m^cisplatin, which was intravenously injected in six HCC cases, was taken up by liver cancers according to first-pass kinetics. When these results are taken together, it is not difficult to assume that HAI using a higher concentration of CDDP can result in greater specific cytotoxic activity against HCC with lower spillover. Based on this concept, a powdered form of CDDP has been marketed in Japan since 2005 that possesses a CDDP concentration higher than any other available CDDP product. Although the safety and efficacy of HAI using powdered CDDP have been confirmed in Phase II studies [10], there are no reports indicating the ideal infusion profile.

This study evaluated the relationship between the tumor response to CDDP-HAI and CDDP dose after normalization with various factors. The results indicate that HCCs can be divided into two groups based on sensitivity or resistance to CDDP. Intriguingly, a higher amount of CDDP may promote rapid tumor growth in CDDP-resistant nodules. A recommended dose of CDDP in HAI and the efficacy of CDDP-HAI are discussed.

## Material and Methods

### Patients

Chemotherapeutic efficacy was evaluated after CDDP-HAI without other therapeutic interventions in 42 HCC nodules in 17 patients from February 2007 to June 2009 in our hospital. Patients suffering from an active malignancy other than HCC were excluded from the study. HAI was the sole therapy applied at the enrollment, as liver transplantation, surgical resection, and radiofrequency ablation were not recommended on the basis of tumor extent and/or functional hepatic reserve or simply because a patient did not prefer those types of treatment. Transarterial chemolipiodolization or chemoembolization was not applied from the trade-off point between functional hepatic reserve and the targeted area volume. Although we definitely presented sorafenib as a promising treatment option to all patients in this stage, the cases analyzed in this study did not prefer to take sorafenib on the basis of the possible adverse events, the cost, and so on. All nodules were radiologically diagnosed as HCC when they fulfilled at least one of the following criteria based on dynamic computed tomography (CT), magnetic resonance imaging (MRI), and/or CT during hepatic arteriography (CTHA), CT during arterial portography (CTAP): (1) a typical hemodynamics of classical HCC, a substantial enhancement during an arterial phase followed by a washout with corona-like peripheral enhancement in an equilibrium phase; or (2) similar characteristics as coexisting nodules that had already been diagnosed as HCC. Because a tumor response was evaluated by quantifying an enhanced area, nodules that did not present substantial arterial supply in dynamic CT/MRI were not enrolled. Finally, the arterial supply was confirmed using CTHA, in which a catheter tip was placed where CDDP would be infused.

Background liver disease was defined by measuring hepatitis B surface antigen (HBsAg), anti-hepatitis C virus (HCV), anti-M2, and antinuclear antibody levels. A habitual alcohol intake of more than 60 g every day was considered alcohol abuse. Nonalcoholic steatohepatitis was diagnosed on the basis of histological findings. When a patient was negative for all above criteria, they were considered not definitive for background liver disease. Although Child-Pugh score was employed for the assessment of functional hepatic reserve based on the clinical assumption of cirrhosis, it is not clear if each case was actually suffering from cirrhosis due to a lack of histological confirmation. Adverse events were evaluated according to the common terminology criteria for adverse events v3.0. Informed consent was obtained from each patient, and the study protocol conformed to the ethical guidelines of the 2008 Declaration of Helsinki as reflected in a prior approval by Niigata University Graduate School of Medical and Dental Sciences Human Research Committee.

### Volumetry using computed tomography

A dynamic study of helical CT scans was performed with 2-mm collimation using a Definition/Sensation 64 (Siemens, Erlangen, Germany) or Aquilion 64 (Toshiba, Tokyo, Japan) scanner. To aid in contrast, 600 mg/kg of iodine contrast medium at a concentration of 370 mg/mL was injected over 30 sec into a peripheral vein, and CT images were obtained at 20 sec after the CT number reached above 200 HU in the aorta, at 30 sec after the initiation of the first phase, and at 180 sec after starting the injection of the contrast medium. The CT scans were performed before (median and interquartile range: 39 and 14–65 days) and after (73 and 55–92 days) HAI. A catheter tip was placed where CDDP would be infused, and CTHA images were obtained at 10 sec after initiating the iodine contrast medium injection at a concentration of 150 mg/mL and a speed of 1 mg/mL over 20 sec via the catheter using a Somatom Sensation 16 (Siemens, Erlangen, Germany) with 2-mm collimation. The images were reconstructed with 2-mm thickness in the arterial, arterial, or portal phase for the volumetry of HCC, CDDP targeting, or the entire liver, respectively.

The liver volumes were manually segmented with a window width of 1000 and a window level of 250. In terms of HCC, a threshold CT number was determined as the mean plus two standard deviations by scanning the surroundings of each nodule. HCC was manually segmented with a window width of (1000 − the threshold) and a window level of (1000 + threshold)/2. The volume between the areas that were defined by manual segmentation was automatically complemented using Aquarius Net Station ver. 1.5 (TeraRecon, Inc., Tokyo, Japan). After manual compensation of the automatic segmentation, the number of pixels was counted in the entirety of the selected areas. With 1 × 1 × 2 mm^3^ for each pixel as a voxel, the entire or target liver volume was calculated, whereas the area of HCC was calculated by dividing the maximal pixel number by 2.

### Hepatic arterial infusion of CDDP

**Figure 1:**
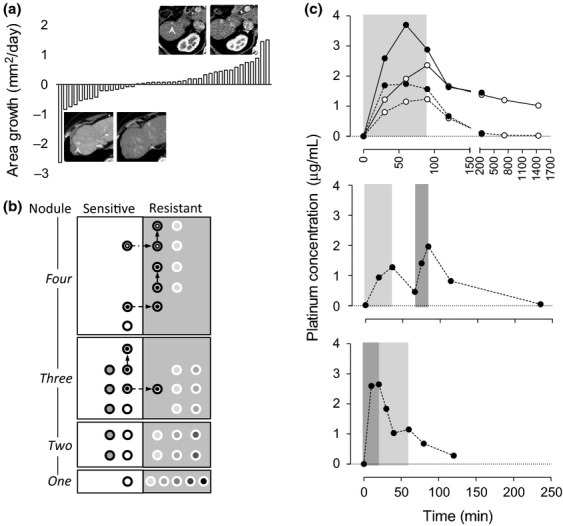
Hepatocellular carcinoma (HCC) response to hepatic arterial infusion (HAI) chemotherapy using cisplatin (CDDP), and the drug distribution during and after HAI. (a) HCC reduction rate (mm^2^/day) of all nodules evaluated in this study during the course of HAI. Fifteen of 42 nodules showed a reduction in size. The maximal area of each nodule was measured in an axial image of dynamic computed tomography (CT). Upper and lower panels are representative CT images in an arterial phase and were obtained before (left column) and after (right column) CDDP-HAI. The upper panel shows that a small nodule in segment 6, R#14-t3-1 in Table 1, enlarged 171% in diameter during 89 days after the treatment. In contrast, a nodule approximately 10 mm in diameter at segment 8, S#10-t1-1 in Table 1, disappeared 84 days after CDDP-HAI (lower panel). The arrowheads indicate each nodule. (b) Behavior of each nodule in association with HAI. Each nodule was separately plotted in a white (CDDP-sensitive) or gray (CDDP-resistant) column based on the number of nodules in an individual case at one to four nodule areas. In each area, the same circle color indicates nodules in one case. Two nodules in a case with three nodules and two nodules in a case with four nodules received HAI twice (a dot within a circle), and one nodule in a case with four nodules received HAI three times (a circle within a circle). The arrows indicate a sensitivity change in each treatment. (c) Serum platinum concentrations in the peripheral (open circles) and/or middle hepatic veins (closed circles) during and after HAI using CDDP. At each point, platinum, concentration was measured as a free form (dashed lines) and/or a total form, including the protein-bound form (continuous lines). In total, 80 mg of CDDP was administered via the proper hepatic artery at the speed of 1 (light gray period) or 2 (dark gray period) mg/min.

The powdered CDDP, IA-call^®^ (Nippon Kayaku Co., Ltd, Tokyo, Japan), was solubilized in saline at a concentration of 100 mg/70 mL just before use. Although CDDP was generally administered with a total dose of 65 mg/m^2^ for the entire liver via the proper hepatic artery, the amount was decreased by 50% or 25% when creatinine clearance (Ccr) was <50 or 25 mg/min/1.73 m^2^, respectively. In addition, when CDDP was infused to a part of the liver, the dose was roughly adjusted by the proportion between the target and nontarget liver volumes based on the inspection of CT images without volumetry. For example, when the right lobe, consisting of 60–70% of the entire liver, was targeted, 60–70% of the 65 mg/m^2^ CDDP dose was administered. Furthermore, when platelet counts were <50,000/mm^3^, the CDDP amount was reduced to half of the maximal dose. The powdered CDDP was infused at the speed of 126 mL/h in all patients except for the cases, in which a pharmacokinetic study of CDDP was performed. On the basis of the treatment efficacy, a successive CDDP-HAI was applied approximately a month after the evaluation using CT. On the other hand, to assay serum platinum concentration, 80 mg of CDDP in liquid form (Randa^®^ Inj.; Nippon Kayaku Co.) was administered via the proper hepatic artery for the entire liver at the speed of 1 or 2 mg/min. In one case, all CDDP was injected at a steady rate of 1 mg/min (Fig. 1c, upper graph), whereas the rate was changed in the middle of HAI from 1 to 2 mg/min or vice versa (Fig. 1c, middle or lower graphs, respectively). When the speed was increased from 1 to 2 mg/min, HAI was halted once for 30 min at the end of the 1 mg/min injection period.

### Serum biochemistry

The platinum concentration was quantified in total and/or a protein-unbound form in the serum collected prior to CDDP administration in addition to 30, 60, 90, 120, 240 min, and 12 and 24 h following CDDP administration from the peripheral vein and/or the middle hepatic vein (MHV). An aliquot of the serum from 5 mL of whole blood was taken and centrifuged at 1500 gravity to filter out proteins larger than 30,000 MW and protein-bound platinum. The platinum concentration measurement was outsourced to Nac Co., Ltd. (Tokyo, Japan) where atomic absorption spectrometry was employed for the measurements. When the injection speed was increased amid HAI, the serum samples were collected prior to HAI in addition to 18, 36, 66, 75, 84, 114, and 234 min following HAI; the measurements were performed on the serum collected prior to HAI in addition to 10-, 20-, 30-, 40-, 60-, 80-, and 120-min postinfusion initiation, when the injection speed was reduced.

HBsAg and anti-HCV antibody levels were detected by a chemiluminescence immunoassay using ARCHITECT HBsAg QT and ARCHITECT HCV (Abbott Japan Co. Ltd., Chiba, Japan), respectively. Antinuclear and anti-M2 antibodies were quantified in sera using a commercial Mesacup ANA ELISA kit (MBL Co. Ltd., Nagoya, Japan) and Mesacup mitochondria M2 kit (MBL Co. Ltd.), respectively. Routine blood biochemistry was measured in the clinical laboratories of our hospital.

Total and *Lens culinaris* agglutinin A-reactive alpha-fetoprotein (AFP) concentrations (ng/mL) in the serum were determined by a liquid-phase-binding assay system (LiBASys; Wako Pure Chemical Industries Ltd., Osaka, Japan), and L3 was calculated as a percentage of *L. culinaris* agglutinin A-reactive species against total AFP. Serum des-γ-carboxy prothrombin (DCP) was measured using an electro-chemiluminescence immunoassay (Wako Pure Chemical Industries Ltd.).

### Statistical analyses

Correlations between HCC response and CDDP dose were quantified by calculating the Spearman correlation coefficient. The repetitive numbers of CDDP-HAI were compared between CDDP-sensitive and CDDP-resistant nodules using extended Fisher's exact test. The differences of metric variables were analyzed between different groups using a Mann–Whitney *U*-test, and were compared using a Wilcoxon matched-pairs signed-rank test to compare between before and after HAI. All analyses were performed using GraphPad Prism 5 (GraphPad Software, Inc., La Jolla, CA), and a two-sided *P*-value <0.05 was considered statistically significant.

## Results

The basic nodule information and their responses to CDDP-HAI are summarized in Tables 1 and 2, respectively. All patients showed performance status 0 except for one case, case 16, which showed performance status 1, both before and after CDDP-HAI. An obvious portal vein or hepatic vein invasion was not observed in this cohort. No substantial lymph node swelling was detected, either, except for one case, case 4, in which para-aortic lymph nodes were diffusely enlarged suggesting massive invasion of HCCs. Distant metastasis was recorded prior to CDDP-HAI in two cases, cases 3 and 4, in lungs. There is no obvious difference in a trend of treatment history between resistant and sensitive groups. The proportion of CDDP-naïve cases and number of sequential HAI were not significantly different between sensitive and resistant cases, either (*P* = 0.48, *P* = 0.61).

**Table 1:** Nodule characteristics

Nodule#	Etiology	Sex	Age	C-P	PH	Prior Tx	Dia	T	AFP	L3	DCP
R#1-t1-1	HCV	F	51	6	Y	TACE, STI	11.5	3	448.0	63.9	45
R#2-t2-2	HCV + AL	M	72	5	Y	OP	14.2	2	175.0	8.6	15
R#3-t1-1	HBV	M	62	7	N	OP, RFA, PEI, TACE	18.2	3	112.0	7.9	58
R#3-t2-1	HBV	M	62	7	N	OP, RFA, PEI, TACE	6.6	3	112.0	7.9	58
R#6-t1-1	NASH	M	84	6	Y	OP, RFA, PEI, TACE	9.4	2	19.9	1.9	81
R#6-t1-2	NASH	M	85	7	Y	OP, RFA, PEI, TACE	9.9	2	29.4	46.5	96
R#6-t2-2	NASH	M	85	7	Y	OP, RFA, PEI, TACE	8.7	2	29.4	46.5	96
R#6-t3-2	NASH	M	85	7	Y	OP, RFA, PEI, TACE	9.6	2	29.4	46.5	96
R#6-t3-3	NASH	M	85	6	Y	OP, RFA, PEI, TACE	15.5	2	150.0	67.4	69
R#8-t1-1	HCV	F	82	6	Y	OP, RFA, PEI, TACE, UFT	11.9	2	56.8	43.9	1645
R#8-t3-1	HCV	F	82	6	Y	OP, RFA, PEI, TACE, UFT	17.9	2	56.8	43.9	1645
R#8-t4-1	HCV	F	82	6	Y	OP, RFA, PEI, TACE, UFT	20.4	2	56.8	43.9	1645
R#8-t5-1	HCV	F	82	6	Y	OP, RFA, PEI, TACE, UFT	9.5	2	56.8	43.9	1645
R#9-t1-1	HBV	M	81	7	N	OP, RFA, PEI, TACE	8.6	3	2.0	NA	11
R#10-t4-2	HCV	F	68	7	Y	RFA, PEI	11.1	2	88.9	2.3	10
R#13-t1-1	ND	M	81	8	N	None	22.6	3	362.0	53.9	222
R#13-t2-1	ND	M	81	8	N	None	20.6	3	362.0	53.9	222
R#13-t3-1	ND	M	81	8	N	None	17.1	3	362.0	53.9	222
R#14-t1-1	NASH	F	77	5	Y	OP, RFA	9.4	2	10.1	3.2	186
R#14-t2-1	NASH	F	77	5	Y	OP, RFA	9.5	2	10.1	3.2	186
R#14-t3-1	NASH	F	77	5	Y	OP, RFA	9.3	2	10.1	3.2	186
R#15-t1-1	HCV	M	80	6	Y	RFA	8.7	2	20.1	2.3	14
R#16-t1-1	HCV	F	76	7	N	OP, RFA, TACE	16.8	2	3.2	LS	400
R#17-t1-1	HCV + AL	M	70	5	N	OP	11.5	3	7.0	LS	23
R#17-t2-1	HCV + AL	M	70	5	N	OP	12.4	3	7.0	LS	23
R#19-t1-2	HCV	F	74	9	Y	TACE	13.7	3	2.2	LS	170
R#19-t2-2	HCV	F	74	9	Y	TACE	13.5	3	2.2	LS	170
S#4-t1-1	HBV	M	60	5	N	OP	10.9	4	42100.0	78.1	11,600
S#4-t2-1	HBV	M	60	5	N	OP	17.6	4	42100.0	78.1	11,600
S#5-t1-1	ND	F	81	6	Y	RFA, PEI, TACE	14.8	3	36.1	51.7	LS
S#5-t2-1	ND	F	81	6	Y	RFA, PEI, TACE	12.8	3	36.1	51.7	LS
S#6-t2-1	NASH	M	84	6	Y	OP, RFA, PEI, TACE	7.1	2	19.9	1.9	81
S#6-t3-1	NASH	M	84	6	Y	OP, RFA, PEI, TACE	13.1	2	19.9	1.9	81
S#6-t4-1	NASH	M	84	6	Y	OP, RFA, PEI, TACE	14.6	2	19.9	1.9	81
S#7-t1-1	AL	M	63	7	N	RFA	9.9	2	5.0	LS	640
S#10-t1-1	HCV	F	68	7	Y	RFA, PEI	12.7	2	101.0	5.7	13
S#10-t2-1	HCV	F	68	7	Y	RFA, PEI	10.0	2	101.0	5.7	13
S#10-t2-2	HCV	F	68	7	Y	RFA, PEI	11.4	2	88.9	2.3	10
S#10-t4-1	HCV	F	68	7	Y	RFA, PEI	10.6	2	101.0	5.7	13
S#12-t1-1	ND	F	81	5	Y	RFA, PEI, TACE, UFT	11.7	3	3.5	LS	343
S#12-t2-1	ND	F	81	5	Y	RFA, PEI, TACE, UFT	8.8	3	3.5	LS	343
S#12-t3-1	ND	F	81	5	Y	RFA, PEI, TACE, UFT	8.4	3	3.5	LS	343

Nodule#, each nodule was assigned as (Resistant/Sensitive)#(Case)-t(Nodule)-(Number of intervention); C-P, Child-Pugh score; PH, if there was a history of cisplatin usage; Prior Tx, therapies applied prior to this study; Dia, diameter (mm); T, tumor factor; AFP, alpha-fetoprotein (ng/mL); L3, fucosylated percentage of AFP (%); DCP, des-γ-carboxy prothrombin (mAU/mL); HCV, hepatitis C virus; AL, alcoholic abuse; HBV, hepatitis B virus; NASH, nonalcoholic steatohepatitis; ND, not definitive. M, male; F, female; Y, yes; N, no; TACE, transarterial chemoembolization; STI, stereotactic irradiation; OP, surgical resection; RFA, radiofrequency ablation; PEI, percutaneous ethanol injection; UFT, combination of tegafur and uracil; NA, not available; LS, less than sensitivity.

**Table 2:** Nodule parameters

Nodule#	BSA	Alb	Ccr	CDDP	CDDP-T	*V*_L_	*V*_T_	Prearea	Postarea	Interval
R#1-t1-1	1.28	3.4	65	50	50	1022.5	1022.5	60.0	85.0	126
R#2-t2-2	1.56	3.5	103	100	100	1209.2	1209.2	44.0	55.0	130
R#3-t1-1	1.71	2.6	67	70	70	924.1	924.1	170.0	270.0	112
R#3-t2-1	1.71	2.6	67	70	70	924.1	924.1	12.5	38.5	122
R#6-t1-1	1.55	3.2	79	70	70	1130.7	1130.7	60.0	70.0	107
R#6-t1-2	1.53	2.8	72	70	50	1181.6	553.2	70.0	80.0	111
R#6-t2-2	1.53	2.8	72	70	50	1181.6	553.2	15.5	65.0	111
R#6-t3-2	1.53	2.8	72	70	50	1181.6	553.2	90.0	155.0	111
R#6-t3-3	1.50	2.9	89	90	90	1127.7	516.2	155.0	235.0	119
R#8-t1-1	1.37	3.5	79	75	50	1136.5	677.8	80.0	135.0	112
R#8-t3-1	1.37	3.5	79	75	25	1136.5	459.2	60.0	65.0	112
R#8-t4-1	1.37	3.5	79	75	50	1136.5	677.8	135.0	150.0	112
R#8-t5-1	1.37	3.5	79	75	50	1136.5	677.8	33.0	55.0	112
R#9-t1-1	1.57	2.4	60	50	50	691.5	691.5	27.0	85.0	134
R#10-t4-2	1.61	1.8	139	90	29	797.4	246.9	55.0	65.0	138
R#13-t1-1	1.71	2.2	73	80	80	970.7	970.7	30.5	160.0	86
R#13-t2-1	1.71	2.2	73	80	80	970.7	970.7	42.0	445.0	86
R#13-t3-1	1.71	2.2	73	80	80	970.7	970.7	9.0	75.0	86
R#14-t1-1	1.77	3.6	76	80	80	1500.6	1500.6	4.6	14.5	89
R#14-t2-1	1.77	3.6	76	80	80	1500.6	1500.6	6.0	13.5	89
R#14-t3-1	1.77	3.6	76	80	80	1500.6	1500.6	17.5	47.5	89
R#15-t1-1	1.40	3.3	62	50	50	794.4	794.4	11.5	75.0	129
R#16-t1-1	1.30	2.5	61	45	45	649.0	649.0	75.0	305.0	159
R#17-t1-1	1.77	3.6	100	100	20	1375.3	380.4	60.0	110.0	133
R#17-t2-1	1.77	3.6	100	100	20	1375.3	380.4	70.0	170.0	133
R#19-t1-1	1.63	2.7	87	60	30	655.9	354.3	65.0	80.0	176
R#19-t2-1	1.63	2.7	87	60	30	655.9	354.3	22.0	27.5	176
S#4-t1-1	1.68	4.1	146	100	100	753.2	753.2	23.5	0.0	44
S#4-t2-1	1.68	4.1	146	100	100	753.2	753.2	115.0	0.0	44
S#5-t1-1	1.38	3.4	62	40	40	1022.7	1022.7	80.0	15.5	87
S#5-t2-1	1.38	3.4	62	40	40	1022.7	1022.7	45.0	39.5	87
S#6-t2-1	1.55	3.2	79	70	70	1130.7	1130.7	23.0	15.5	107
S#6-t3-1	1.55	3.2	79	70	70	1130.7	1130.7	110.0	90.0	107
S#6-t4-1	1.55	3.2	79	70	70	1130.7	1130.7	125.0	75.0	107
S#7-t1-1	1.69	3.9	78	75	75	1027.3	1027.3	125.0	45.5	119
S#10-t1-1	1.55	2.5	98	90	70	759.9	531.4	70.0	0.0	84
S#10-t2-1	1.55	2.5	98	90	70	759.9	531.4	38.5	21.5	84
S#10-t2-2	1.61	1.8	139	90	61	797.4	550.5	21.5	0.0	138
S#10-t4-1	1.55	2.5	98	90	20	759.9	228.5	90.0	55.0	84
S#12-t1-1	1.31	3.7	52	50	50	1074.0	1074.0	75.0	0.0	154
S#12-t2-1	1.31	3.7	52	50	50	1074.0	1074.0	31.0	0.0	154
S#12-t3-1	1.31	3.7	52	50	50	1074.0	1074.0	19.0	0.0	154

Nodule#, each nodule was assigned as (Resistant/Sensitive)#(Case)-t(Nodule)-(Number of intervention); BSA, body surface area (m^2^); Alb, serum albumin concentration (g/dL); Ccr, creatinine clearance (mL/min/1.73 m^2^); CDDP, total infusion dose of cisplatin, CDDP-T, cisplatin-dose administered to target liver volume; *V*_L_, the entire liver volume; *V*_T_, target liver volume; prearea, maximal axial area of HCC prior to HAI; postarea, maximal axial area of HCC after HAI.

### HCC response to CDDP-HAI

The area reduction or growth of each nodule is plotted in Figure 1a. CDDP-HAI was repeated two and three times for four nodules in two patients, cases 6 and 10, and for one nodule in one patient, case 6, respectively (Fig. 1b). Representative CT images of the nodule indicating resistance or sensitivity to CDDP are presented in Figure 1a. Multiple nodules in the same liver revealed the same response to CDDP except for one case, case 6, in which one nodule was resistant, while the other three were sensitive as indicated in the nodule area of four in Figure 1b. No resistant nodule became sensitive over the course of repeated CDDP-HAI, whereas three sensitive nodules acquired resistance (Fig. 1b dotted arrows). The maximum diameter and area in an axial section of each HCC was calculated using the dynamic CT images taken before and after each HAI with an interval of 113 ± 30 days. The average diameter of a HCC was 12.4 ± 3.9 mm, and 15 nodules presented a size reduction at the speed of 0.42 mm^2^/day (median, 0.063–2.6), while the other 27 HCCs grew at the rate of 0.28 mm^2^/day (0.031–1.5). There were no significant differences between the sensitive and resistant groups in terms of gender (*P* = 0.52), age (*P* = 0.35), tumor factor (*P* = 0.50), tumor diameter (*P* = 0.66), AFP (*P* = 1.00), L3 (*P* = 0.27), DCP (*P* = 0.58), and Child-Pugh score (*P* = 0.20). During and after CDDP-HAI, the extrahepatic lesions including lymph nodes gradually progressed in cases 3 and 4.

The influence of CDDP-HAI on functional reserve and quality-of-life is summarized in Table 3. The difference of median values (after − before treatment values) of white blood cell counts, platelet counts, serum albumin, creatinine, prothrombin time, total bilirubin, and MELD score were −140, −0.2, 0, 0.04, 0.01, 0.2, and 0, respectively, and was significantly different in creatinine and total bilirubin values (*P* = 0.013 and *P* = 0.0036, respectively). Child-Pugh score was not significantly changed (*P* = 0.069). Although mild ascites developed after the treatment in four cases, cases 3, 10, 13, and 16, it was temporal events and easily controlled using diuretics. Hepatic encephalopathy was developed in none of the cases. In terms of quality-of-life, subjective symptoms were evaluated using the Japanese version of the Support Team Assessment Schedule (STAS-J) [11]. In the first category of pain and other symptom, the deterioration from 0 point to 2 points was recorded only in one case, case 2, while 1 point in two cases, cases 1 and 16, improved to 0 point after the treatment. Similarly, in the second category of patient anxiety and awareness of their prognosis, only one case, case 17, showed worsening after the treatment from 0 to 1 point, while four cases, cases 1, 3, 16, and 12, improved 1 or 2 points after the treatment. In other cases, no differences were observed in both categories before and after CDDP-HAI. Although mild appetite loss and nausea were developed in three cases, cases 1, 2, and 16, an intravenous injection of metoclopramide hydrochloride could easily relieve the symptom.

**Table 3:** Treatment influence

Nodule#	WBC	Plt	Alb	Crt	PT	T Bil	MELD	ST1	ST2
R#1-t1-1	4920−4850	13.5−9.4	3.4−3.3	0.80−0.82	0.98−0.97	0.9−1.0	6-6	1-1	0-0
R#2-t2-2	4390−3440	10.0−9.8	3.5−3.1	0.81−0.78	1.08−1.12	0.8−1.0	7-8	0-0	2-0
R#3-t1-1	3010−2780	8.5−9.4	2.6−2.8	0.93−0.89	1.20−1.20	1.5−2.1	10-1	0-2	0-1
R#3-t2-1	3010−2780	8.5−9.4	2.6−2.8	0.93−0.89	1.20−1.20	1.5−2.1	10-1	0-2	0-1
R#6-t1-1	7090−7040	9.1−10.6	3.2−3.3	0.76−0.89	1.17−1.23	0.9−1.0	8-9	0-0	0-0
R#6-t1-2	5830−6230	11.7−11.3	2.6−3.0	0.73−0.87	1.14−1.07	1.1−1.0	8-7	0-0	0-0
R#6-t2-2	5830−6230	11.7−11.3	2.6−3.0	0.73−0.87	1.14−1.07	1.1−1.0	8-7	0-0	0-0
R#6-t3-2	5830−6230	11.7−11.3	2.6−3.0	0.73−0.87	1.14−1.07	1.1−1.0	8-7	0-0	0-0
R#6-t3-3	4950−8320	12.0−16.8	2.6−2.6	0.96−0.88	1.11−1.12	1.1−1.3	8-9	0-0	0-0
R#8-t1-1	4430−4380	8.1−7.9	3.5−3.3	0.69−0.73	1.11−1.02	1.1−1.0	8-7	0-0	0-0
R#8-t3-1	4430−4380	8.1−7.9	3.5−3.3	0.69−0.73	1.11−1.02	1.1−1.0	8-7	0-0	0-0
R#8-t4-1	4430−4380	8.1−7.9	3.5−3.3	0.69−0.73	1.11−1.02	1.1−1.0	8-7	0-0	0-0
R#8-t5-1	4430−4380	8.1−7.9	3.5−3.3	0.69−0.73	1.11−1.02	1.1−1.0	8-7	0-0	0-0
R#9-t1-1	4370−4240	10.6−8.4	4.3−4.3	1.09−1.13	0.99−1.05	0.7−0.9	7-8	0-0	0-0
R#10-t4-2	4070−3080	11.2−8.6	1.8−2.8	0.51−0.52	1.19−1.17	1.1−1.5	9-10	0-0	0-0
R#13-t1-1	2080−2520	9.3−5.8	2.2−2.7	0.98−1.04	1.25−1.27	0.9−1.2	9-9	0-0	0-0
R#13-t2-1	2080−2520	9.3−5.8	2.2−2.7	0.98−1.04	1.25−1.27	0.9−1.2	9-9	0-0	0-0
R#13-t3-1	2080−2520	9.3−5.8	2.2−2.7	0.98−1.04	1.25−1.27	0.9−1.2	9-9	0-0	0-0
R#14-t1-1	3480−3120	11.8−11.3	3.6−3.5	0.62−0.60	1.13−1.08	1.2−1.0	8-7	0-0	0-0
R#14-t2-1	3480−3120	11.8−11.3	3.6−3.5	0.62−0.60	1.13−1.08	1.2−1.0	8-7	0-0	0-0
R#14-t3-1	3480−3120	11.8−11.3	3.6−3.5	0.62−0.60	1.13−1.08	1.2−1.0	8-7	0-0	0-0
R#15-t1-1	2510−3730	7.6−8.5	3.3−3.3	0.87−0.90	1.15−1.22	1.6−1.9	10-11	0-0	0-0
R#16-t1-1	4620−6170	22.5−14.5	2.5−2.1	0.41−0.52	1.16−1.63	0.7−0.7	8-2	1-1	0-0
R#17-t1-1	5030−4190	16.1−11.4	3.6−3.7	0.77−0.97	1.13−1.09	0.6−0.5	8-7	0-0	0-1
R#17-t2-1	5030−4190	16.1−11.4	3.6−3.7	0.77−0.97	1.13−1.09	0.6−0.5	8-7	0-0	0-1
R#19-t1-2	1680−2330	2.1−3.1	2.7−3.0	0.50−0.49	1.57−1.58	3.0−3.3	16-16	0-0	0-0
R#19-t2-2	1680−2330	2.1−3.1	2.7−3.0	0.50−0.49	1.57−1.58	3.0−3.3	16-16	0-0	0-0
S#4-t1-1	5190−3116	14.7−16.5	4.1−3.4	0.66−0.77	1.05−1.06	0.5−0.6	7-7	0-1	0-1
S#4-t2-1	5190−3116	14.7−16.5	4.1−3.4	0.66−0.77	1.05−1.06	0.5−0.6	7-7	0-1	0-1
S#5-t1-1	5560−4400	7.8−8.2	3.4−3.0	0.88−1.33	1.16−1.24	0.9−1.3	8-13	0-0	0-0
S#5-t2-1	5560−4400	7.8−8.2	3.4−3.0	0.88−1.33	1.16−1.24	0.9−1.3	8-13	0-0	0-0
S#6-t2-1	7090−7040	9.1−10.6	3.2−3.3	0.83−0.89	1.17−1.23	0.9−1.0	8-9	0-0	0-0
S#6-t3-1	7090−7040	9.1−10.6	3.2−3.3	0.83−0.89	1.17−1.23	0.9−1.0	8-9	0-0	0-0
S#6-t4-1	7090−7040	9.1−10.6	3.2−3.3	0.83−0.89	1.17−1.23	0.9−1.0	8-9	0-0	0-0
S#7-t1-1	5120−4980	6.5−7.0	3.9−3.2	1.03−1.00	1.35−1.38	2.9−3.0	14-14	0-0	0-0
S#10-t1-1	3760−2370	10.9−8.0	2.5−2.8	0.51−0.51	1.18−1.17	1.5−1.8	10-10	0-0	0-0
S#10-t2-1	3760−2370	10.9−8.0	2.5−2.8	0.51−0.51	1.18−1.17	1.5−1.8	10-10	0-0	0-0
S#10-t2-2	3760−3080	10.9−8.6	1.8−2.8	0.51−0.52	1.19−1.17	1.1−1.5	9-10	0-0	0-0
S#10-t4-1	3760−2370	10.9−8.0	2.5−2.8	0.51−0.51	1.18−1.17	1.5−1.8	10-10	0-0	0-0
S#12-t1-1	5060−4210	12.4−12.9	3.7−4.5	0.72−0.82	1.08−1.00	0.7−0.6	7-6	0-2	0-0
S#12-t2-1	5060−4210	12.4−12.9	3.7−4.5	0.72−0.82	1.08−1.00	0.7−0.6	7-6	0-2	0-0
S#12-t3-1	5060−4210	12.4−12.9	3.7−4.5	0.72−0.82	1.08−1.00	0.7−0.6	7-6	0-2	0-0

Nodule#, each nodule was assigned as (Resistant/Sensitive)#(Case)-t(Nodule)-(Number of intervention); WBC, a number of white blood cells per volume of blood (/mm^3^); Plt, a number of platelets per volume of blood (×10^4^/mm^3^); Alb, albumin concentration in serum (g/dL); Crt, creatinine concentration in serum (mg/dL); PT, prothrombin time in international normalized ratio; T Bil, total bilirubin concentration in serum (mg/dL); MELD, a model for end-stage liver disease with modification from United Network for Organ Sharing (http://www.mayoclinic.org/meld/mayomodel6.html); ST1 and ST2, points of Japanese version of the support team assessment schedule in categories 1 and 2, respectively.

### Platinum distribution and time course during and after HAI

As shown in the upper graph of Figure 1c, both the free- and total-platinum concentrations quickly increased following CDDP-HAI and were substantially higher in the MHV than in the periphery. Remarkably, free-platinum concentration in the MHV was approximately stable through HAI, while the concentration gradually increased toward the end of HAI in the periphery leading to the equilibration of the concentration between the MHV and the periphery. With a similar equilibration, total platinum slowly disappeared from the serum on a daily basis, while free platinum quickly decreased to an undetectable level in <2 h following the end of HAI. The total-platinum concentrations in the periphery at 4, 12, and 24 h after the initiation of HAI were 1.38, 1.20, and 1.02 μg/mL and presented a strict correlation (*r*^2^ = 0.99) suggesting that the platinum level would return to the background approximately 80 h after finishing HAI. Although the absolute values vary among cases, the free-platinum concentration is higher when CDDP was administered at the rate of 2 mg/min in comparison with an infusion at 1 mg/min in the same individuals (Fig. 1c, middle and lower graphs).

### CDDP-sensitive nodule responded in association with the administration dose at the local level

**Figure 2:**
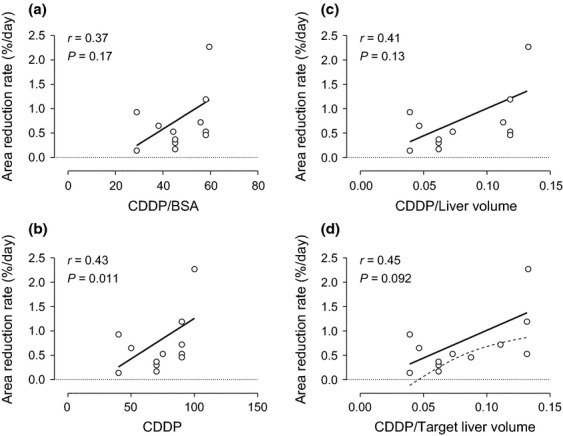
Correlations between reduction rate of hepatocellular carcinoma after hepatic arterial infusion (HAI) chemotherapy and cisplatin (CDDP) dose normalized by various factors in CDDP-sensitive nodules. The area reduction rate (%/day) in 15 CDDP-sensitive nodules was not significantly correlated with the total amount of CDDP administered (*P* = 0.11, *r* = 0.43), CDDP dose normalized with body surface area (*P* = 0.17, *r* = 0.37), or CDDP dose normalized with the entire liver volume (*P* = 0.13, *r* = 0.41) as shown in b, a, or c, respectively. (d) The CDDP dose presented the largest correlation coefficient of 0.45 and tended to correlate with the reduction rate after standardization with the target liver volume (*P* = 0.092, *r* = 0.45). Continuous or dotted lines revealed a linear regression with the least sum-of-square or 95% confident interval, respectively.

In 15 CDDP-sensitive nodules, there was no correlation between the administration dose of CDDP and the area reduction rate of HCC either with normalization or without normalization by body surface area as shown in Figure 2a and b, respectively (*P* = 0.17, *r* = 0.37 or *P* = 0.11, *r* = 0.43, respectively). Because there was no CDDP-dose dependency on tumor reduction rate from a systemic point of view, the correlation was evaluated at the local level. Although there was no significant correlation between HCC reduction rate and CDDP dose after normalizing with the entire liver volume (Fig. 2c, *P* = 0.13, *r* = 0.41), there was a tendency toward positive correlation by dividing the CDDP dose with the target liver volume (Fig. 2d, *P* = 0.092, *r* = 0.45). These results suggest that CDDP-sensitive nodule size reduction is a function of local CDDP concentration. A 95% confidence interval indicates that an infusion of 0.05 mg CDDP for every mL of target liver volume is enough to determine whether each nodule is sensitive to CDDP. The average CDDP doses for target liver volume were 0.077 ± 0.025 and 0.080 ± 0.038 mg/mL in resistant and sensitive cases, respectively, and were not significantly different from each other (*P* = 0.65).

### Larger amounts of CDDP promoted a rapid tumor growth in CDDP-resistant nodule

**Figure 3:**
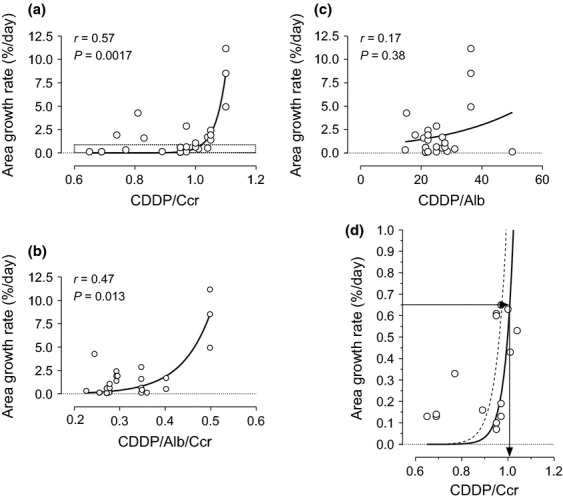
Correlations between the growth rate of hepatocellular carcinoma after hepatic arterial infusion (HAI) chemotherapy and cisplatin (CDDP) dose normalized by various factors in CDDP-resistant nodules. The area growth rate (%/day) in 27 CDDP-resistant nodules was significantly correlated with CDDP dose after normalization by creatinine clearance (a, *P* = 0.0017, *r* = 0.57) but not after normalization using serum albumin concentration (c, *P* = 0.38, *r* = 0.17). Although CDDP after standardization with both albumin and creatinine clearance also indicated a significant correlation (b, *P* = 0.013, *r* = 0.47), the probability and correlation coefficient are lower than a simple normalization with creatinine clearance. (d) An enlarged graph corresponding to the indicated area of figure 4a (a dotted box) that shows an area growth rate of 0.65%/day, which equals to a doubling time of 90 days, is plotted against a CDDP dose of approximately 1 mg for every creatinine clearance. A continuous or dotted line revealed a linear regression with the least sum-of-square or 95% confident interval, respectively.

As shown in Figure 1c, free platinum disappears from the blood stream over several hours after finishing HAI, while total platinum is slowly excreted from the body over a couple of days or more. Albumin is a major protein that CDDP binds, and urine is a main excretory route. Thus, we evaluated efficacy of HAI in later phases by adjusting the CDDP dose with albumin, Ccr, or both. Although no significant correlation was observed in CDDP-sensitive nodule after each adjustment (data not shown), the HCC growth rate in the CDDP-resistant group was positively correlated with CDDP dose in an exponential fashion after normalization by Ccr (Fig. 3a, *P* = 0.0017, *r* = 0.57) or with both albumin and Ccr (Fig. 3b, *P* = 0.013, *r* = 0.47). Because a simple Ccr normalization revealed a higher probability and correlation coefficient, and because a normalization with albumin abrogated the correlation (Fig. 3c, *P* = 0.38, *r* = 0.17), the clinical significance was further evaluated after normalization with Ccr. When tumor doubling time without therapeutic intervention is assumed to be 90 days, which is equivalent to a 0.65%/day growth rate, a dose of CDDP higher than 1 mg/Ccr may enhance the tumor growth rate, making it faster than the natural growth rate as shown in Figure 3d.

## Discussion

Because overall HCC survival is definitely defined by not only anatomical tumor extent but also the functional liver reserve as an integrated staging system can stratify overall survival quite well [2,3], an anticancer treatment should be less toxic to the hepatocytes especially in cases with deteriorated functional hepatic reserve. To date, sorafenib is the only treatment option that clearly revealed survival benefits in HCC at advanced stages based on a large-scale-randomized prospective study such as SHARP study [12]. However, we have experienced difficulties to make patients with similar characteristics of the cohort in this study keep taking sorafenib and follow a stable disease. One reason for the difference between SHARP study and our experience would be a difference of a patient profile. The age in our cohort, 73.5 ± 9.4 years, is significantly older than 64.9 ± 11.2 years of SHARP cohort (*P* = 0.0012, *t*-test). Especially for the patients with Child A, which is recommended for sorafenib administration, the median age in our cohort is 80 years. A majority of patients in SHARP study was relatively naïve for any type of treatments. Only 19%, 6%, or 29% received resection, radiofrequency ablation, or transarterial chemoembolization in SAHRP, respectively, while 53%, 63%, or 47% had a history of each treatment in our cohort, respectively. These differences may draw a different outcome. On the other hand, Kim et al. [13] recently reported that HAI may be a better alternative for HCC at advanced stages than transarterial chemoembolization, which revealed survival advantage in randomized prospective studies and a meta-analysis for unresectable HCC [14–17]. CDDP-HAI offers relatively higher killing effects against HCC and lower hepatocyte toxicity. Powdered CDDP can be administered at a high concentration of 100 mg/70 mL, which is higher than any other CDDP available in liquid form (0.5 or 1 mg/mL in Japan or USA, respectively). The overall response and a 1-year survival rate were reported to be 33.8% and 67.5%, respectively, in a Phase II study enrolling 63.5% of stage IV-A cases [10]. The major adverse events were grade 3/4 anorexia and grade 3/4 thrombocytopenia, which were observed in 22.5% and 25% of the cases, respectively. In this study, no severe adverse events were recorded in both systemic and liver reserves, and in quality-of-life. CDDP-HAI may have a potential to be a promising alternative treatment option for HCC at advanced stages from the both points of efficacy and toxicity profile. On the other hand, a number of HAI and CDDP dose that were normalized by target liver volume were not significantly different between sensitive and resistant HCC. Multiple nodules arising in an individual tended to present the same profile after CDDP-HAI. Taken together, the sensitivity to CDDP-HAI is supposed to be a tumor-inherent characteristic at least with a dose applicable in clinic. In this regard, ideally, the sensitivity to CDDP should be gauged, and CDDP-HAI should be limited to those sensitive to CDDP. Unfortunately, however, so far there is no surrogate marker available for this purpose.

A correlation between CDDP dose/(target liver volume) and HCC reduction rate in CDDP-sensitive nodules revealed that 0.05 mg for every mL of target liver volume is sufficient to judge whether the nodule is sensitive or resistant to CDDP. At the same time, this study indicated that the CDDP amount should be lower than a dose that would promote faster growth of HCCs. In terms of HCC doubling time, there is a large variation with a range in the average of 136–204 days. Correlations with initial tumor diameters were reported in cases with an HCC size smaller than 50 mm [18,19]. Okada et al. [20] reported a shorter average doubling time of 112 days and a median of 80 days for 27 recurrent HCCs <20 mm in diameter. Because our cohort consists of the nodules with an even smaller diameter of 12.4 mm on average, a 90-day doubling time was employed in this study. Based on this hypothesis, the recommended dose of CDDP is between 0.05 mg/(target liver volume) and 1 mg/Ccr when there are no data indicating CDDP sensitivity. For example, in a case with 50 mL/min of Ccr after adjustment to 1.73 m^2^ of body surface area, the CDDP dose administered should be <50 mg. As long as the target liver volume is <1000 mL, 50 mg of CDDP is enough to explore nodule sensitivity in the entire liver. When the nodules are judged to be sensitive to CDDP after the first HAI, the CDDP dose may be increased based on other factors such as leukocytopenia and/or deterioration of kidney function. If Ccr is 30 mL/min, the maximal CDDP dose should be 30 mg. In this case, the target should be restricted to a liver volume of <600 mL. If the entire liver volume is larger than 600 mL, it would be better to perform HAI via a hepatic artery that feeds <600 mL involving a majority of the HCC in the first HAI. In such a case, CTHA with a catheter tip that is placed where HAI will be performed is very useful to determine actual target liver volume. If a favorable result is obtained, the target area can be expanded by increasing the administration dose of CDDP.

The maximum tolerated and recommended doses of CDDP-HAI were reported as 80 and 65 mg/m^2^, respectively [10]. Therefore, CDDP-HAI is usually initiated at a dose of 65 mg/m^2^. Although 65 mg/m^2^ and our criterion were defined in a completely different way, practically the maximum recommended dose was similar between both cases as long as patients have a regular body size and normal kidney function. For example, if body surface area is 1.73/m^2^, 65 mg/m^2^ gives 112 mg/body. Because creatinine clearance should be over 100 mL/min with normal kidney function, the maximum dose of over 100 mg/body is expected from this study. As body size is smaller, the dose is getting relatively larger in our setting.

Carcinogenesis and cancer treatment are two sides of the same coin [21]. In fact, carcinogens and anticancer drugs highly overlap. Immortalized endocervical cells selected for resistance to CDDP are malignantly transformed [22]. Alkylating-like agents are classic carcinogens, and at the same time, they are classic anticancer drugs [23]. Radiation is both an effective anticancer therapy and a carcinogenic agent as reflected in the Chernobyl accident [24,25]. It is not difficult to assume that the preferential killing of drug-sensitive cancer cells simultaneously selects for resistance that can be accompanied by tumor progression through a multifactorial phenomenon related to both genetic and epigenetic pathways [26–29]. Furthermore, the restoration of the heterogeneous populations of T cells and the reestablishment of T-cell immunocompetence is a slow and frequently incomplete process after T-cell depletion in the context of cytotoxic anticancer therapy [30]. In contrast, several recent lines of evidence suggest that a low-dose chemotherapy may display positive immunological effects during cancer therapy by depleting immunosuppressive cells such as CD4^+^ CD25^+^ regulatory T cells and myeloid-derived suppressor cells [31]. In addition, chemotherapy-induced cell death may release tumor antigens that could be uptaken by antigen presenting cells, processed, and presented to naïve T cells [32]. Those effects could enhance latent antitumor immune response or synergize with a tumor vaccine. Although it is still under debate whether low-dose chemotherapy actually can improve overall survival, it is strongly suggested that HAI with high-dose CDDP cannot reduce a tumor burden in CDDP-resistant nodules and may even lead to unfavorable results. Furthermore, even a higher tumor response achieved by HAI in metastatic liver tumors could not improve overall survival when compared with systemic chemotherapy in colorectal cancer [33]. Taken together with a significant increase of serum creatinine and total bilirubin values after HAI, it is advisable to avoid a high-dose CDDP-HAI unless CDDP sensitivity is evident.

CDDP affected CDDP-sensitive or CDDP-resistant nodules in distinctive ways. A higher impact probability was obtained after normalization with the target liver volume or Ccr, respectively, suggesting that CDDP concentration or CDDP clearance plays a key role in each condition. Achieving higher impacts on CDDP-sensitive nodules while evading growth promotion in CDDP-resistant nodules require both a higher concentration of free CDDP in the liver and a rapid disappearance of total CDDP from the body. It was reported that clearance of platinum compounds after a short-term (4–15 min) intravenous infusion of CDDP is triphasic, with a distribution half-life of 13 min, elimination half-life of 43 min, and terminal half-life of 5.4 days [34]. The elimination and terminal half-lives are consistent with the disappearance rate of free- and total-platinum in this study, respectively. The distribution half-life suggests that HAI targeting a relatively small area will easily exceed a tissue distribution speed and saturate the tissue with CDDP as in the hepatic vein under HAI (Fig. 1c). The concentration of free platinum in the MHV depends on the CDDP injection rate but not on total amount of CDDP. Thus, the total amount of CDDP acceptable from the point of adverse effects should be injected in a short period via the hepatic artery targeting HCC. Because data suggesting an appropriate CDDP injection speed to obtain the best cancer effects is not available, a study clarifying the time dependency of the HCC response to CDDP-HAI should be conducted.

## Conclusion

This study suggests that the sensitivity to CDDP is intrinsically defined in HCC and cannot be improved by a dose escalation and/or repetition of HAI in most cases. To avoid inducing unfavorable adverse events, a lower dose sufficient to confirm CDDP sensitivity should be employed during the initial CDDP-HAI. Standardizations are recommended to define CDDP dose using the target liver volume and creatinine clearance. Because Japanese cohort may be biased in such as age and extensive treatment history, further efficacy evaluations of CDDP-HAI at stages beyond locoregional treatment indications should be conducted to show the improvement of overall survival and treatment response as well as the improvement of sensitivity by appropriate injection speeds and dose.
